# Supporting Indigenous Family Caregivers of Children with Life-Threatening and Life-Limiting Illness in One Canadian Province: Healthcare Providers’ Perspectives

**DOI:** 10.3390/children12070895

**Published:** 2025-07-07

**Authors:** Jill M. G. Bally, Meridith Burles, Amaya Widyaratne, Victoria A. Spurr, Heather Hodgson-Viden, Roona Sinha

**Affiliations:** 1 College of Nursing, University of Saskatchewan, Saskatoon, SK S7N 2Z4, Canada; arw524@mail.usask.ca (A.W.); vas028@usask.ca (V.A.S.); 2 St. Thomas More College, University of Saskatchewan, Saskatoon, SK S7N 5A2, Canada; mburles@stmcollege.ca; 3 College of Medicine, University of Saskatchewan, Saskatoon, SK S7N 5E5, Canada; heather.hodgsonviden@usask.ca (H.H.-V.); roona.sinha@usask.ca (R.S.)

**Keywords:** family nursing, Indigenous healthcare, childhood life limiting illnesses, childhood life threatening illnesses, qualitative research, childhood chronic illness

## Abstract

**Background:** Indigenous peoples in Canada endure lasting effects of colonialism including racism, marginalization, and social, political, and geographic inequities resulting in disproportionate rates of disease and inequitable healthcare. Indigenous infants and children in Canada experience a high incidence of birth complications and illnesses, and families are often left to navigate the care of their child with limited resources. Accordingly, improved, culturally responsive and safe healthcare is needed to enhance child outcomes and optimize family well-being. **Purpose and Methods:** A qualitative study was conducted by our research team including family members of Indigenous children with serious illnesses, a Cultural Advisor, researchers, and pediatric clinicians. In one component of the study, we explored pediatric healthcare providers’ (HCPs) experiences of caring for Indigenous families of children with serious illness. A purposive sample of 19 pediatric healthcare providers took part in semi-structured qualitative interviews or one focus group. The data were analyzed thematically to identify common experiences and priorities for improved supportive healthcare. **Findings:** Five themes were identified representing insights from pediatric HCPs, with a focus on barriers to effective healthcare at the interpersonal, institutional, and system levels for seriously ill Indigenous children and their families. Ideas for enhancing the social and cultural responsiveness and safety of supportive healthcare were identified. **Implications and Conclusions:** The findings offer valuable insights and strategies for HCPs to support holistic, comprehensive, and culturally safe and responsive healthcare.

## 1. Introduction

Indigenous peoples are integral members of Canadian society, yet they face an array of social and health disadvantages due to historical and ongoing colonialism [1,2]. For instance, direct and indirect impacts of colonialism have contributed to disparities among Indigenous children and their families, including disproportionate rates of life-threatening and life-limiting illnesses and higher rates of infant and childhood mortality [3,4,5,6]. To close the gap in health disparities between Indigenous and non-Indigenous infants and children in Canada, it is essential that the legacy of colonization and ongoing effects are acknowledged, and efforts are made to implement The Truth and Reconciliation Commission (TRC) Calls to Action [7] into healthcare practice, thus enhancing provision of culturally responsive and safe care for Indigenous children and their families [8].

Indigenous peoples are the first occupants of what is now Canada, and include three groups identified by the Constitution Act, First Nations, Inuit, and Métis peoples [2]. The Indigenous population consists of 1.8 million peoples who constitute 5% of the total Canadian population [2]. Moreover, from 2016 to 2021, the Indigenous population grew at twice the rate of non-Indigenous, with children under the age of 15 representing one-quarter (25.4%) of the Indigenous population [2,9]. Despite the relatively young Indigenous population in Canada, health disparities persist, and Indigenous children experience disproportionately worse health outcomes due to the legacy of colonization and ongoing impacts on social determinants of health [1,3]. As such, efforts are needed to ensure access to appropriate and timely healthcare and support for Indigenous families with children affected by serious illnesses and health complications.

### 1.1. Colonialism, Social Determinants, and Health Disparities Among Indigenous Peoples’ in Canada

According to Allan and Smylie (2015) [10], the health and well-being of Indigenous peoples must be understood within the context of colonization and the persistent colonial influences in Canadian society. Historically, colonial practices included assertion of control over Indigenous peoples’ traditional territories, implementation of the reservation system, and dispossession of language and cultural traditions through forced assimilation and the residential school system [11]. These actions threatened the physical and psychosocial well-being of Indigenous people due to diminished access to traditional foods and restrictions on participation in, and dissemination of cultural traditions and maintenance of kinship networks [12]. In addition, the residential school system was a colonial assimilation strategy that involved removal of children from their families and placement in schools aimed at assimilating Indigenous children into British settler society and eradicating Indigenous cultures and communities [7]. Numerous harms endured at residential schools resulted in personal and intergenerational trauma that has been a central contributor to the social and health inequities facing many Indigenous individuals, families, and communities [10,13]

Furthermore, the lasting implications of colonialism for Indigenous peoples in Canada are highly evident with respect to social determinants of health. For instance, low income and socioeconomic status, substandard housing, food insecurity, and barriers to healthcare all play a role in disproportionately poor health outcomes [13,14]. Additionally, Indigenous people face systemic discrimination that is embedded in the policies and practices of institutions, along with interpersonal racism, contributing to further social and health inequities [15]. Therefore, colonialism has had direct and lasting implications for the health and well-being of Indigenous peoples in Canada.

The inequitable social-environmental context experienced by many Indigenous peoples in Canada can impact the daily lives of infants and children, making them especially vulnerable to health disparities [4,16]. Life-limiting illness (LLIs) and life-threatening illness (LTIs) disproportionately affect Indigenous infants and children, including birth complications, congenital conditions, tuberculosis, and cancer [11,17,18,19,20]. For instance, Indigenous newborns have a higher probability of being preterm and malnourished, which are associated with increased health issues in infancy and early childhood [20]. Tuberculosis (TB) is a public health emergency in several Indigenous communities in Canada [18], with Indigenous children having an increased risk of developing TB and a high incidence of complications such as TB meningitis [18]. Additionally, despite comparable rates of cancer diagnosis, Indigenous children have considerably lower life expectancies when diagnosed with cancer in comparison to non-Indigenous children [19]. Thus, Indigenous infants and children frequently face poor health that requires healthcare intervention.

### 1.2. Indigenous Families’ Challenges with Accessing Healthcare

Despite the increased prevalence of health disparities among Indigenous infants and children, Indigenous families often experience challenges when accessing healthcare and support. For example, many Indigenous families face difficulties with accessing healthcare services related to geographic, transportation, financial, and systemic barriers [5,21,22]. Indigenous families often do not have access to health information and services close to where they live, necessitating travel for long distances to obtain appropriate services for their children [5]. However, the availability of transportation can be very limited, complicating access to healthcare and generating stress for Indigenous families [5]. Additionally, although healthcare in Canada is universally available, financial barriers are common when accessing acute care services due to the costs of travel, parking, and charges associated with walk-in clinic, and medications [6]. Additionally, the costs of health services are also problematic for Indigenous families living in poverty, leading to decreased means for rent, food, and diapers. Recent research has indicated that Indigenous parents can be subject to racism and discrimination when accessing healthcare services, which results in families feeling humiliated, distressed, and less likely to seek healthcare in the future [5,6,22]. Furthermore, negative interactions within the healthcare system lead to mistrust and difficulties building rapport and constructive relationships with healthcare providers (HCPs). Accordingly, actual and anticipated discrimination in the healthcare system is a significant barrier to the well-being of Indigenous families navigating care for their seriously ill child.

### 1.3. Calls to Actions for Healthcare Providers and Pediatric Cultural Safety

The TRC of Canada included 94 Calls to Action that acknowledged the wide-ranging harms caused by colonialism and promotes the process of reconciliation [7]. Seven of the Calls to Action are associated with health and highlight healthcare providers’ responsibility to provide culturally responsive and safe care for Indigenous peoples. Recommendations include acknowledgement of the colonial mechanisms and related health disparities, efforts to work with Indigenous peoples to create goals for improving health outcomes, incorporation of Indigenous healing practices into patient care, and implementation of cultural safety training for healthcare providers [7]. A culturally safe approach can enable healthcare providers in addressing Indigenous peoples’ concerns and diminish the incidence of maltreatment, thus contributing to improved health outcomes and supports [6,23]. To guide equitable healthcare practices, cultural safety can be seen as an “outcome based on respectful engagement that recognizes and strives to address power imbalances inherent in the healthcare system. It results in an environment free of racism and discrimination, where people feel safe when receiving healthcare” [24] (p. 13). Grounding care in cultural safety supports self-reflective practice and awareness of power differences, ensuring healthcare is person-centred rather than practitioner or institutionally driven [25]. Culturally safe healthcare is especially important when working with Indigenous children as they are developing their cultural identity, and any form of disrespect may be detrimental to this process [26]. Despite recognition of the importance of culturally safe healthcare and support services, little is known about HCPs’ experiences with providing care for Indigenous children with LLIs and LTIs or how they understand effective, culturally safe healthcare aimed at supporting Indigenous families when caregiving for a seriously ill infant or child.

## 2. Materials and Methods

The research reported herein was developed by a Patient and Family-Oriented research (POR) team. A POR approach was adopted wherein families with related experiences and a Cultural Advisor played a significant role on the research team and in the development of the research process [27]. Patient-Oriented Research involves patients or family members with experiences of a health issue in the identification of priorities for research, which values their knowledge and capacity to ensure meaningful research [27]. Other members of the research team included a registered nurse researcher/educator, health sociologist, pediatric palliative care specialist, pediatric oncologist/hematologist, and students in health-related studies. Considerable consultation occurred with families, the Cultural Advisor, and team members to determine the appropriate theoretical grounding for the research process [27,28]. Initially, the research team engaged family members who had firsthand experience of caregiving for children with health conditions to ensure the research was relevant and in line with their priorities. Emphasis was placed on collaboration and involvement of several advisors in the development of the research including recruitment, data collection, data analysis, and knowledge translation, to the extent that they wanted to participate. Specifically, consultation took place with local support organizations, and family and cultural advisors to decide on the research agenda and to consider priorities. Discussions included personal experiences, cultural beliefs and practices, and issues that impact families who are navigating care for a child with a LLIs or LTIs. Together, it was decided that the research would comprise two parts: One component of the study engaged family caregivers, while the second component included pediatric HCPs; the latter is described in this manuscript. The overall purpose was to improve understanding of Indigenous family caregivers’ experiences of negotiating care of children with LLIs and LTIs in a Canadian mid-western province, with the aim to inform enhanced cultural responsiveness and safety in healthcare and support services. The specific objectives of this qualitative research were to: (1) Explore HCPs’ perceptions of Indigenous family caregivers’ experiences and challenges of navigating care and support for a child with an LLI or LTI; and, (2) identify strategies and suggestions for culturally safe, family care and support at the institutional, system, and community levels. The questions guiding the research were: (1) What are the healthcare experiences and challenges of Indigenous families of children with LLIs and LLIs?; and, (2) what do HCPs feel would enhance existing care and support services to be more culturally meaningful and helpful to Indigenous families’ well-being and coping?

### 2.1. Sample and Setting

A purposive and snowball sample of HCPs who engage with Indigenous families as part of their current role in pediatric healthcare were recruited including registered nurses, social workers, pharmacists, respiratory and music therapists, physicians, and a healthcare assistant. The HCPs had experience providing care for children aged birth to 14 years diagnosed with an LLI or LTI and their fathers, mothers, and/or other family caregivers that self-identified as Indigenous (First Nations, Inuit, or Métis) and were urban, rural, or remote residents from the mid-western Canadian province where the research was conducted.

The sample was recruited from one urban maternal-child hospital where infants and children with suspected LLIs and LTIs are referred for diagnosis and treatment (105–130 new diagnoses per year). Participants were recruited through the distribution of brochures to relevant HCPs who worked in the pediatric care setting and an associated support organization (i.e., Ronald McDonald House). If interested, individuals were asked to use workplace email addresses or telephone numbers to contact one of the researchers, who have several years of qualitative health research experience, for more details and to arrange participation in an individual interview or focus group discussion. Once an HCP expressed interest, a research team member with experience in recruitment contacted them to provide additional details and completed informed consent procedures.

### 2.2. Ethical Considerations

Prior to beginning the study, ethical and operational approval were sought through University’s Behavioural Research Ethics Board and the local Health Authority, respectively. Potential participants were informed of the voluntary nature of participation, and that withdrawal of their data was possible at any time prior to completion of data analysis. Informed consent also included assurance that the data collected during this study would be reported in an anonymized format for conferences and publications so that participants could not be identified. Informed consent was obtained from each participant by email prior to the individual or focus group interview. The consent forms were stored on a password-protected computer, kept in a locked cabinet in the first author’s work office. Additionally, the first three authors had access to the raw data. An experienced transcriptionist was given the audio-recordings and/or field notes to transcribe, but no participant contact information was included. The transcriptionist signed a confidentiality form prior to receiving the recordings. The researchers and the transcriptionist ensured that all data were de-identified by removing names from transcripts and were stored in a separate locked cabinet from the consent forms and participant contact information in the first author’s office. The research team including the family and cultural advisors, and physicians, had access to the de-identified data for the purpose of data analysis and interpretation.

### 2.3. Data Collection

One focus group was conducted virtually with 11 participants along with individual, virtual interviews with eight participants to generate data about the context of care and logistical issues related to cultural safety in pediatric care and support services. The first two authors (J.M.G.B., M.B.), who are experienced health researchers, used a semi-structured interview guide developed by the research team to support the individual and focus group interactions [29,30]. Along with professional experiences, participants were asked about the supports available to Indigenous families and the possibility of enhanced integration of cultural practices into healthcare as a way to improve care and support, as this was a priority identified by the research team in collaboration with the family and cultural advisors. Examples of questions included: What experiences have you had with caring for Indigenous children and their families?; and, how do you think that we could enhance existing services or programs, or create new ones to ensure that Indigenous families are well supported in caring for their child? With informed consent, all interviews were audio-recorded and completed via Zoom (*n* = 9) based on the HCPs’ preference, although a variety of options were provided for the interviews (in-person, virtual, focus groups, and individual) to offer maximal flexibility and convenience, and reduce barriers for participation. Demographic details, field notes to document the context and any non-verbal communication, and memos that were written to document developing analysis were also collected. Data collection and analysis were deemed sufficient when researchers perceived that a good understanding of the topic had been gained [30,31] based on depth and breadth of responses.

### 2.4. Data Analysis

The data were transcribed verbatim by an experienced transcriptionist and analyzed thematically using Braun and Clarke’s [32] reflexive method of analysis. Data analysis began with the first three authors (J.M.G.B., M.B., A.W.) becoming familiar with the data through engagement with the transcripts. The first and third authors read, reread, made notes about, and consistently met to code the data. Coding of the data was completed manually using Microsoft Word documents, tables, and a variety of colours to sort, organize, and differentiate data, ideas, codes, and ultimately themes. Both authors discussed the coding as it progressed to ensure analytical understanding and a systematic and thorough process. Once coding was considered complete, the data were compiled for each code [32]. Phase 3 involved addressing the research questions through generation of themes whereby codes were clustered into similar ideas or “candidate themes” [32] (p. 35). Through collaboration and in-depth familiarity with the coded data, the authors (J.M.G.B., A.W.) moved to Phase 4 to complete development and review of the candidate themes during which five key themes were finalized. In Phase 5, the first three authors (J.M.G.B., M.B., A.W.) met again and refined the description of the finalized themes to ensure that the ‘story’ for each theme was clearly and accurately told, and that the naming of the themes was appropriate [32] (see Table 1).

**Figure 1:**
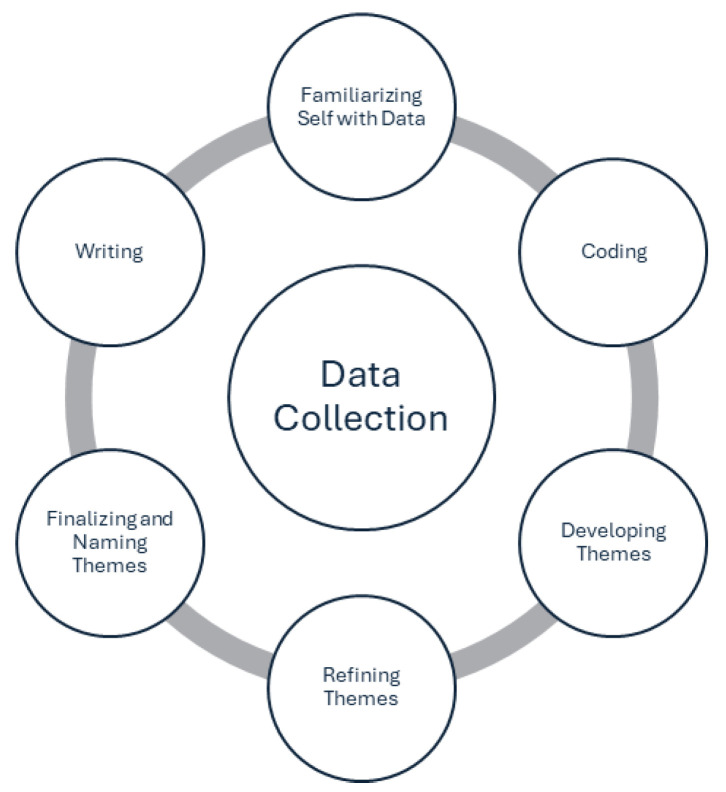
Process of data analysis (adapted from Braun and Clarke, 2021 [32]).

The process of data analysis took place over several months and culminated in presentation and discussion of the findings with the majority of the research team. Last, Phase 6 involved writing the research report presented herein [32] (see Figure 1).

### 2.5. Rigour

Various steps were taken during the research to ensure high quality, trustworthy data and findings. Credibility was assured by audio-recording and verbatim transcription of the interviews and focus groups to ensure accuracy, and dependability was developed using an audit trail to document all research processes [33]. Credibility and confirmability of the findings resulted from collaboration with family members, clinicians, and the Cultural Advisor on the research team, and a rigorous analytic process involving several readings of the data, documentation of emerging themes, and consideration of team members’ interpretations [33]. The inclusion of multiple HCPs’ perspectives enhanced representative credibility by capturing a diversity of viewpoints. The authors sought transferability by using purposive sampling to obtain a heterogenous sample and ensuring a rich description of the context within which the research was completed [31].

## 3. Findings

The findings are situated in the context of one mid-western Canadian province which is made up of 774 incorporated municipalities (454 urban; 296 rural; 24 northern municipalities) with 18% of the province’s population residing in rural locations [34]. The province comprises six treaty areas (2, 4, 5, 6, 8 and 10), is part of the Homeland of the Métis Nation, and has 74 First Nations of a variety of linguistic groups; approximately 37,000 people live in the rural and northern communities of the province of whom about 87.4% self-identify as Indigenous [34]. Healthcare for residents in the province’s northern rural and remote communities is challenging and limited as “there are no permanent doctors, and doctor visits are few and far between, if not delayed for weeks due to poor weather conditions that prevent travel” [34] (p. 36). As a result, seeking healthcare can be costly both financially and personally such as being separated from family, work, and one’s cultural, language and traditional supports. Many of the children and families cared for by the HCPs in this study lived in the province’s northern rural and remote communities and traveled for care to the urban children’s hospital.

A total of 19 HCPs participated in this phase of the research (see Table 2). The participants were diverse in terms of their roles and experiences in providing care to Indigenous families of children with LLIs and LTIs; however, limited characteristics of the sample are presented given the small geographic area from which the participants were drawn to ensure confidentiality. Through analysis of the data, five themes were developed, reflecting the experiences and perceptions of diverse pediatric HCPs.

### 3.1. Facing and Mitigating Access Barriers

HCPs reflected on the various challenges that they felt were faced by some Indigenous families when accessing healthcare services and caring for their child, as well as effective strategies for overcoming barriers to healthcare. Challenges included transportation and travel issues, technology barriers, poor-quality housing, and inferior access to medication. For example, one HCP highlighted the frequent need to travel for care, stating that:

*“Often the nearest hospital or healthcare centre is quite far away. [Families] end up having to travel long distances. Even just for blood work or for someone to keep an eye—put an eye on them.”*

Accordingly, the lack of healthcare services within many families’ communities created logistical challenges that required navigation. Participants also identified the various transportation supports in place, as well as new ideas for mitigating barriers, such as funding programs, travel and accommodation coordination, as well as respite supports. For families living in reserve communities, travel for healthcare was often provided; however, there were issues reported by some participants with the coordination of community-based transportation, which could pose an issue with being on time for appointments for some families. As one HCP stated:

*And so that’s really important to be aware of I know I’ve heard before that sometimes there are some hiccups, especially with transportation … I’ll try hard, but I can’t promise that they are going to give me the taxi voucher that a family needs to get here. It doesn’t matter. The struggle is real.*

On the other hand, some support for overcoming barriers to healthcare was found to be effective. For instance, one participant reiterated the importance of Jordan’s Principle [35], a policy aimed at ensuring resolving jurisdictional funding barriers such that Indigenous children do not have unmet needs, stating:

*I have to say that one thing that has been very helpful for some of our patients is when you’ve been able to make a specific ask using Jordan’s Principle, and often kind of services that, like I never would have thought would be covered, we do, we are able to get covered.*

In raising this point, this participant and others highlighted the role that HCPs played in securing essential resources for families through this initiative.

Additionally, HCPs described the effects of the COVID-19 pandemic in alleviating or exacerbating access issues for Indigenous families. One participant reflected on the challenges encountered:

*“I have to say that for the last two years, it’s been more of a struggle with, kind of COVID, and having a lot of restrictions with who’s able to come in for healthcare.”*

Meanwhile, another participant reported on how the circumstances shifted healthcare delivery in a beneficial way, noting:

*I think, interestingly, COVID has been a big benefit for a lot of kids of Indigenous background further from larger cities, but also other individuals living in more remote areas. Because it’s opened up more willingness to do Telehealth care. We make it so that when things come up, families can then stay in their home community that’s also benefit.*

As this example illustrates, some participants described possibilities that existed for working with families to make adaptations to limit barriers. Nonetheless, access to technology and other resources could also be limiting factors.

### 3.2. Centrality of Family and Community

The participants recognized the centrality of family and community bonds when supporting and engaging with Indigenous families. The HCPs highlighted the various community members and family dynamics that can constitute a patient’s core circle such as single and blended families, grandparent caregivers, as well as Elder and community member involvement, all of whom are important to acknowledge and consider throughout the child’s care process. In recognizing the importance of extended family and community members’ involvement in the healthcare of the child and family, one participant described efforts to support such engagement:

*We did have a patient who had probably something like 60 family members. They were able to kind of come through and meet with them in the hospital setting. The were there in the last few hours, which I think was very helpful and supportive for the [immediate] family.*

Other important considerations when supporting Indigenous patients and their families were discussed including reducing caregiving tasks for single caregivers, allocating family spaces in health centres, and asking questions about patient’s support circle. One participant captured the potential benefit of community supports for ensuring comprehensive and timely healthcare:

*The other thing is the band office [for families from reserve communities]. The band office is really good too for support. We call the band office a lot saying right—after we get permission from the family—this is the situation; how can we help the family? What can you do to help us? And maybe it’s arranging transportation, maybe it’s reminding the patient to have their appointment? Maybe it’s getting their medications delivered to their house. So, I think the other thing that we’ve done is actually called the Chief a couple times saying, ‘here’s the situation, we need some help’. Like, how are you going to help this family, and they’re really good at stepping up and helping families.*

Additionally, participants called attention to the need for consideration of how parental/caregiver experiences can influence the child’s care process such as caregiver stress, parental addiction/risk of relapse, and parental education. When recollecting the experiences of one family whose child had complex medical needs, a participant shared the immense stress that was experienced:

*These families, they recognize the benefit of not having to do dialysis every night, but they also live a life of, I guess, smoldering stress because that risk of either, you know, a severe infection resulting in sepsis and the need for hospitalization [of their child] versus, you know, always the risk of rejection because, you know, their immunosuppressive status is not adequate. It’s hard. There’s a lot of stress in there for sure.*

Another HCP related the challenges with addictions that factors into their plan of care with some families, sharing that:

*We do a lot with addictions. So, ensuring that the parents themselves are healthy and safe. And, making some safety plans with—if we know that because of the complexity of your child [child’s health needs], you’re at an increased risk of maybe relapsing, right? And, how are we going to ensure that doesn’t happen?*

Accordingly, participants’ stories demonstrated how care of the child was provided within the broader context of family and community relationships and circumstances.

### 3.3. Providing Culturally Safe and Relevant Care

Healthcare providers recognized the importance of considering culturally safe and relevant practices in every step of the family’s care processes. Such considerations included respectful engagement and support for cultural engagement within the healthcare setting. For instance, one participant explained how they promote communication about families’ cultural and/or spiritual affiliations, recalled:

*One thing I say is, do you belong or are you a part of any religious or cultural communities and that usually opens up … so that’s kind of, the line I use in my assessment. And I don’t think a lot of social workers ask that, that’s not in our general assessment, but I started putting it in and I find it opens up things.*

Additionally, some HCPs in this study recognized that some Indigenous families may want to utilize traditional healing practices and engage with cultural conceptions of health during their child’s healthcare journey. Thus, participants discussed practices in place for facilitating cultural engagement and novel ideas for including culturally relevant practices within pediatric healthcare settings. For instance, the involvement of Elders, ceremonial spaces in hospitals, and the translation of English resources into Indigenous languages were successful initiatives. As well, the importance of being open to discussion of culture and traditional practices was highlighted by one HCP who stated:

*I think holistic kind of medicine often comes into the conversation in terms of treatment, ways to treat various conditions. I have a lot of patients from Indigenous background that will ask about different, medicinal things, is there anything else we can do activity-wise or accessing herbal medication? and things like that comes into the conversation, too.*

Thus, cultural engagement was fostered by HCPs through openness to dialogue about different approaches to health and healthcare. Importantly, another HCP explained their efforts to adjust to Western biomedical language with the aim of being more respectful and culturally safe:

*I started trying to use the term ‘healthy living’ instead of lifestyle modifications based on you know, these kinds of one-off conversations that they have where one of the physicians from Manitoba, Allison Dart, she’s done a lot of research in terms of First Nations’ care. One of the things that kind of hit her one time is, you know, the term ‘lifestyle modification’, and somebody came back and retorted, you’ve been modifying my life for centuries, I really don’t care to have that. So, taking on the term of trying to say ‘healthy living’—I’ve tried to incorporate that and avoid that other kind of term.*

In sharing this recognition, this participant emphasized the need to account for the legacy of colonialism in interactions with families, with the aim to avoid perpetuation of paternalistic attitudes within healthcare. Relationship building was also a key component of cultural safety, as described below.

### 3.4. Inadequacies of the Healthcare System

Participants described the inadequacies of our current healthcare system when addressing the needs of Indigenous pediatric patients and their families. Despite recognition that improvements have been made, participants noted that there remains vast potential for growth and advancement to improve the care experiences of Indigenous families of children with LLIs and LTIs at the systems level. Inadequacies included an unclear electronic record system, issues with community/urban coordination, and health benefit system errors.

Amidst such challenges, HCPs also discussed the value of interdisciplinary team members such as health educators and social workers, and the various barriers that prevent these members from their optimal function on the healthcare team. Such barriers stemmed from role confusion among healthcare team members and limited positions for culturally relevant support staff. As one HCP said,

*I think some of the staff feel like they should be more involved in having this or that discussion or involved in discharge planning. But it’s unclear for everyone what their role is, or maybe their role in that situation itself.*

Another participant discussed the organizational challenges related to families’ attendance of appointments in urban settings saying:

*Half the time [families] get here the coordination of their accommodations and transportation is all wrong, we’re fixing it and it makes families, because then they’re stuck, they’re at the hotel waiting. So, last week, the band did one for me [organized travel for a family appointment], the hotel was full, so the family got to the hotel, they called me and they’re like, ‘what do we do, now we’re coming from far away, we’re stuck at this hotel, but it’s full’, and so now they have to move hotels. So, it’s just this, it’s, and it’s something that our team here has tried to communicate, that it’s very unfortunate that it’s just, it’s not a smooth system and it makes people frustrated, and it really has deferred people from coming to medical appointments.*

Some participants reported that such coordination issues strained relationships between HCPs and families, which could negatively influence the child’s care. Along with the challenges related to organization of care, there were significant challenges identified with human resources as pointed out by one participant:

*The other thing is in, this is just for this local children’s hospital, but we have a social worker for every ward and sometimes two. When it comes to the First Nations and Métis Health Educators, there’s four positions for all the hospitals. And so, this is just my personal opinion, but the Health Authority has kind of done this commitment to Indigenous supports, but there’s no more positions being added. And then what happens is the educators are getting burnt out and leaving or going on sick leave. There’s a high turnaround there or they’re frustrated and overwhelmed and we’re trying to work with them. Then the lines get blurred of whose role is to do what, because [general social workers are] also involved, because they’re too busy.*

Thus, support providers such as social workers and First Nations and Métis Health Educators played a crucial role in filling in gaps within the healthcare system to ensure families were well supported.

### 3.5. Communicating Effectively and Building Trusting Relationships

Amidst various system and community-level challenges, participants recognized their crucial role in fostering supportive relationships and communications. There were various factors that the participants felt went into building trusting relationships with Indigenous patients and their families, a crucial aspect to providing care. Strategies for effective communication regarding child’s care process were discussed, such as using teach-back methods, practicing care training at appointments, and adjusting treatment plans based on parental/familial needs. Accordingly, there were some circumstances where additional efforts were essential to ensure that families were supported in caring for their child because the available information was not necessarily clear. For instance, one participant explained:

*It happens a lot, where the family will be like, ‘we just don’t get this or we’re confused about this’, [and] I am able to go back to the doctor and say, okay, the family is very confused on this, can we simplify it? And so, sometimes, again lots of it, and most of the time, I would say 99% of time it’s a miscommunication or not understanding.*

Similarly, another participant discussed supportive teaching, stating:

*You know, we do gauge, and we do a lot of teach-back methods and stuff like that. And we actually often times have them—the patients admitted to the hospital and then make sure that the family’s, you know, during the training and after the training, are observed for a period of time doing the dialysis, for example, that occurs overnight, you know, doing the correct sterile setup, doing the correct sterile connect and disconnect. You know, families are never going to be 100 percent, but I would say the majority of the time they [are] really ready to go. Because they’re just like, OK, you know what? We can do this. We’ve been able to do this. I think we’ve been successful with that kind of health education.*

Additionally, important considerations for building trusting relationships with Indigenous patients and their families were discussed such as maintaining consistent interactions with families, having one-on-one conversations, and being more present. One HCP recalled:

*Just simple interactions. I think sometimes in healthcare, we overthink, or we always have to go in with a mission or a reason. And we don’t, so I’m really trying to regularly just visit with families, especially with families that have little kids and they just need to talk to an adult. And I even try to be, try not to talk about medical stuff sometimes, I try to discuss ‘what are you doing when you’re having fun?’ and usually that leads to something, [I] try to relate that way.*

Participants also talked about HCPs’ attitudes and behaviours, and how these can affect relationships and trust with families of pediatric patients, such as how personal biases shown by HCPs can lead to parental hesitancy to inform HCPs of conditions. One participant emphasized the need to improve relationship building efforts, recalling:

*But most of the nurses, they’re just outside of that room, sitting outside of the room, just watching the patient and not really connecting with them inside. And so maybe education is needed, too, for lots of our nurses. It could be some training of some sort or just providing an idea about what our Indigenous patients’ unique needs are and what we could do to support them and provide specific culturally rooted care.*

While some educational initiatives exist, participants suggested additional education and learning opportunities provided by Indigenous peoples would be beneficial to promoting understanding, as would collecting the Elders and Indigenous families’ perspectives about culturally safe care.

## 4. Discussion

Existing literature has highlighted the health disparities among Indigenous children, along with the challenges experienced by Indigenous families, capturing the urgent need for culturally safe care in the pediatric context [5,6,22,23,27]. The purpose of this research was to obtain a deeper understanding of the needs and priorities for culturally responsive and safe care and support identified by pediatric HCPs, while family caregivers’ perspectives are described elsewhere (Burles et al., in preparation [36]). A thematic analysis of the data [32] resulted in five themes representing insights from pediatric HCPs, focusing on barriers to effective healthcare at the interpersonal, institutional, and system levels for seriously ill Indigenous children and their families. Participants also shared initiatives that they viewed as working well for addressing families’ needs, as well as ideas for enhancing the social and cultural responsiveness of supportive pediatric healthcare.

In this study, Indigenous families of children with LLIs and LTIs were reported to *Face Access Barriers* when seeking pediatric healthcare including transportation and travel issues, technology barriers, poor-quality housing, and inferior access to medication. While supports for overcoming such barriers exist, participants recalled frequent challenges that had broader implications for care and relationships with HCPs. Similarly, in a narrative literature review exploring barriers to healthcare access for adult Indigenous peoples, the authors identified several factors including geography [37]. Specifically, the authors identified challenges related to living in rural and remote reserve communities such as scarce access to a full range of HCPs, lack of year-round road access necessitating evacuation by air in emergent circumstances, and limited internet infrastructure limiting online access to multiple medical resources [37]. Integrating findings from multiple studies, the National Collaborating Centre for Indigenous Health (NCCIH) [38] stated that, throughout Canada, there are critical limitations to healthcare access for Indigenous peoples given geographic barriers. As well, existing efforts have yet to ensure reliable access to technology and Internet in some regions, which limits access to healthcare and information [37]. While the existing evidence is not exclusive to Indigenous families with children who have LLIs and LTIs, there are similar outcomes including longer wait times for healthcare, delayed healthcare, separation of families for care, lengthy and expensive travel, and sometimes even foregoing care [38].

Participants in the current study also described the effects of the COVID-19 pandemic as both alleviating and exacerbating issues related to accessing pediatric healthcare. The finding that the pandemic supported use and acceptance of Telehealth involving a virtual medical appointment, particularly for rural and remote families, was unique to this study as most studies have emphasized the additional challenges created by the COVID-19 pandemic. On the other hand, pandemic-related challenges included isolation from important familial relations and community members (e.g., Elders, Knowledge Keepers), and traditional healing practices and ceremonies [38] which were also noted by the HCPs in this study.

The identification of various supports in place, as well as new ideas for supports intended to mitigate identified barriers to accessing care, highlight valuable steps for ensuring holistic care and support of Indigenous families. For example, HCPs identified existing funding programs such as Jordan’s Principle, as well as the critical importance of travel and accommodation coordination and respite supports, emphasizing that such forms of supportive care must be made available to Indigenous families to reduce some of the challenges that were faced when seeking care for their children. While Jordan’s Principle [35] is well known in Canada and is used to overcome jurisdictional issues between government bodies that lead to a lack of timely care for Indigenous children, it is not always easily implemented and may require HCPs or support providers to initiate on the families’ behalf. However, continuing efforts are being made via the Jordan’s Principle Child-First Initiative to work towards reducing jurisdictional issues and inequities in relation to funding [3]. As well, community-led initiatives offer promise to improve support of families in their own communities, thereby reducing and even mitigating challenges related to accessing pediatric healthcare; for example, one community in Manitoba, Canada has developed a community-based programme titled My Child, My Heart for First Nations children living with complex medical needs and their families which may be acceptable and feasible or adapted through co-designed research for use in other communities.

Additionally, the *Centrality of Family and Community Bonds* were seen as crucial when supporting and engaging with Indigenous families in this study. The HCPs identified various community members and family dynamics that can constitute a pediatric patient’s core circle, often extending beyond immediate family. While these findings reflect the context and families being cared for by participants in this study, other authors substantiate the importance of considering the interconnectedness of Indigenous peoples to larger family and community networks [39], reinforcing the importance of creating space for their inclusion in care and support processes. HCPs in this study also identified various parental/caregiver characteristics and experiences that may influence the child’s care process such as caregiver stress, parental addiction/risk of relapse, and the need for supportive and appropriate caregiver education. Similarly, a scoping review focused on family-centred interventions used to support early childhood well-being in several countries including Canada [40]. McCalman et al. [40] highlighted the importance of advocating for the social determinants of health when supporting children and families. This included advocating for supports and resources related to mental health and substance use, child access and custody, and enhancing maternal knowledge and skills through individual and group teaching [40].

Additionally, a Canadian study by Carrier et al. [16] explored Indigenous philosophies and implementation of Indigenous ways of knowing to improve healthcare specifically for neonates in the neonatal intensive care unit and their families, also identifying the need to recognize the social, cultural, and historical context in which families navigate care of their child. For example, the authors discussed the possibilities of significant emotions, fear, anxiety related to personal demands, and uncertainty in the care setting, which can reflect current experiences and prior challenges faced in one’s lifetime [16]. Similarly to the participants in the current study who emphasized the importance of providing consistent HCPs for each family and time spent communicating with children who have LLIs and LTIs and families, Latimer et al. [8] provided specific strategies that can reduce uncertainty and related emotional experiences. For instance, these authors suggested avoidance of stigmatizing questions that are “racialized and culturally insensitive” about drugs and alcohol when not pertinent to the reason for admission and care [8] (p. 109). Alternatively, participants in this study recommended open communication that allowed for cultural and spiritual beliefs to be shared.

In the current study, important considerations when supporting Indigenous patients and their families were identified such as reducing caregiver tasks for lone parent caregivers, allocating family spaces in health centres, and asking questions about patient’s circle of support and cultural preferences. Similarly, McCalman et al. [40] found that one of the key strategies of such interventions was the need to promote community connectedness. These authors identified the critical importance of garnering community engagement in supporting children and their families to support family togetherness and to act as advocates for enhancing culturally safe healthcare [40]. Furthermore, the authors of the studies included in their scoping review recommended that early childhood programmes be developed and used as a central community space to not only offer healthcare but also to meet social support needs of community members [40].

*Providing Culturally Safe and Relevant Care* was highlighted by the HCPs in this study and included the importance of considering culturally appropriate practices in every step of the family’s care process. The HCPs shared that some Indigenous families may want to utilize traditional healing practices and draw upon Indigenous ways of knowing in their child’s illness and healthcare journey. Novel ideas for improving culturally relevant practices were suggested, as well as the appreciation of practices that are already in place. These culturally relevant practices included the involvement of Elders within the healthcare setting, access to smudging supplies and ceremonial spaces in hospitals, time for ceremony in healthcare spaces, and translation of English resources to region-specific Indigenous languages such as Cree and Michif. Existing literature supports the critical need to provide care that is community-driven including “culture and lore,” and the choice to receive health education in the preferred language [40] (p. 13). Similarly, in a qualitative study conducted in British Columbia, Canada, Pilarinos et al. [41] explored experiences of racism and recommendations for enhancing cultural safety in healthcare with Indigenous adults, describing the need for spaces that were welcoming through the inclusion of Indigenous cultures and traditional health practices.

While there were many positive steps being taken to improve pediatric healthcare for Indigenous families and their children who had LLIs and LTIS, the HCPs in this study recognized the need for broader efforts to address remaining *Inadequacies of the Healthcare System*. These areas for growth included issues with the electronic record system, community/urban coordination, and the health benefit system. Additionally, participants discussed factors that prevent interdisciplinary team members, such as health educators and social workers, from making valuable contributions to the optimal functioning of the healthcare team. These factors include role confusion among the healthcare team and limited availability of culturally relevant support staff. Similarly, the participants in studies by Auger, Howell, and Gomes [42] and Pilarinos et al. [41] highlighted the ongoing need for funding to support enhancement of human health resources including mandatory cultural competency training for healthcare staff and increased Indigenous-run support services and leadership. More specifically, the NCCIH [38] suggested an emphasis on community ownership and authority over healthcare services to encourage healthcare learner placements in rural and remote northern and on-reserve Indigenous communities, as well as recruitment and retention strategies aimed at HCPs in Indigenous communities.

*Communicating Effectively and Building Trusting Relationships* in this study encompassed the various factors that are needed to build trusting relationships with Indigenous patients and their families. This was seen by the HCPs as a crucial aspect of providing care. Strategies for effective communication regarding child’s care process were discussed such as using teach-back methods, practicing care training at appointments, and adjusting treatment plans based on parental needs. Important considerations were identified for building trusting relationships with Indigenous patients and their families such as maintaining consistent interactions with families, having one-on-one conversations, and having a greater presence. The ideas shared by participants relate to findings arising from a qualitative study conducted by Latimer et al. [8] involving four First Nations and 188 children, youth, and their parents along with community members to explore their perspectives and healthcare experiences. The team developed the “First Approach (Family, Information, Relationship, Culturally Safe-Space, and Two-Eyed Support)” [8] (p. 18) which acts as a guide for HCPS to use when developing relationships with Indigenous peoples including children and families. In alignment with the HCPs in this study, the emphasis of First Approach is on relationship building to support improved healthcare for Indigenous families and their children. Similarly, Carrier et al. [16] stated that nurses should utilize opportunities to build relationships with parents and facilitate open and honest communication. Specifically, these authors advocate for a trauma-informed approach that can foster successful relationship-building and effective communication through active listening, validating concerns, and a non-judgmental approach [16]. Furthermore, participants in this study spoke about the value of Indigenous HCPs in improving trust amongst Indigenous families and the healthcare team.

Additionally, education and understanding of the legacy of colonialism were highlighted by some participants in the current study as essential to avoiding personal biases and developing respectful, trusting relationships which can compromise communication with families. Similar findings were also reported by Carrier et al. [16] which speaks to a need for HCP education provided by Indigenous peoples such as Elders and Indigenous families who could offer insight into the meaning of culturally responsive and safe care. Existing literature also emphasized collaboration as a key dimension of culturally safe care characterized by shared decision-making and attention to the emotional and spiritual needs of children and families through a strength-based and holistic approach [16]. As such, this research offers insight into specific strategies for cultural safety advocated by the participants, which can be adapted and expanded upon in other healthcare settings.

## 5. Conclusions

The findings from this research enhance understanding of the context of healthcare and support provision for Indigenous children diagnosed with a LLI or LTI and their families in one Canadian province, offering valuable insights for HCPs to enhance holistic, comprehensive, and culturally safe healthcare to Indigenous children and their families. Ultimately, the experiences shared by pediatric health and support providers highlighted the necessity of individual, institutional, and system level efforts to promote cultural safety in pediatric care and support for families navigating the complexities of their child’s illness and care. For example, strategies for improved access to pediatric healthcare included the use of Telehealth, particularly for those in rural and remote communities, and the need for improved access to Jordan’s Principle. Furthermore, participants reinforced the need for creating space for inclusion of family and community members (e.g., Elders, Knowledge Keepers) in healthcare and the importance of recognizing the significant parental/caregiver experiences that may influence a child’s care including stress, addition/risk of relapse, and the need for supportive caregiver education in region-specific Indigenous languages. It was also recognized that there is a need for funding to support human health resources including Indigenous healthcare staff and cultural competency training. The need for a trauma-informed approach to support trusting relationship building was raised along with methods for effective communication such as using the teach back method, one-on-one conversations, and having a greater, more consistent presence. As such, these findings and those generated through qualitative research with Indigenous family caregivers (reported elsewhere) can inform development of support activities and initiatives that promote holistic, culturally safe, and responsive healthcare.

## Figures and Tables

**Table 1:** Analytical development of themes.

Quotations	Examples of Codes	Final Themes
“Often the nearest hospital or healthcare centre is quite far away. So they end up having to travel long distances. Even just for blood work or just for someone to take an eye on them. Keep an eye—put an eye on them”.	Having transportation issues for rural families. Having health centres further away. Having no pharmacy in community can create access issues.	Facing and Mitigating Access Barriers
“I have to say that one thing that has been very helpful for some of our patients is when you’ve been able to make a specific ask using Jordan’s Principle, and often kind of services that, like I never would have thought would be covered, we do, we are able to get covered”.	Using Jordan’s Principle for respite care. Using Jordan’s Principle for treatment devices. Having Jordan’s Principle advocates is good support.	
“We did have a patient who probably something like 60 family members, were able to kind of come through and meet with them. Kind of be even in the hospital setting, kind of in the last few hours, which I think was very helpful for the family and supportive for the family.”	HCPs should understand importance of extended family. Having extended family involvement can be a benefit to care. Having number of family members present at end of life.	Centrality of Family and Community
“Also, we do a lot with addictions. So ensuring that the parents themselves are healthy and safe. And making some safety plans with —if we know that because of the complexity of your child, you’re at increased risk of maybe relapsing, right. And how are we going to ensure that doesn’t happen?”	Considering parental addiction and prevention. Considering increased risk of relapse due to circumstances. Using unhealthy coping habits. Recognizing ongoing trauma and adjustment during treatment process.	
“I think holistic kind of medicine often comes into the conversation in terms of treatment, ways to treat various conditions. I have a lot of patients from indigenous background that will ask about different, medicinal things, is there anything else we can do activity wise or accessing herbal medication and things like that comes into the conversation too.”	Acknowledging the holistic dimensions of chronic illness. Utilizing cultural facilities more. Elders helping make end of life decisions. Talking about holistic medicine/treatment. Being more open to traditional healing methods. Asking family directly about cultural practices/preferences.	Providing Culturally Safe and Relevant Care
“And so, it happens a lot, where the family will be like, we just don’t get this or we’re confused about this, I am able to go back to the doctor and say, OK, the family is very confused on this, can we simplify it. And so, sometimes, again lots of it, and most of the time, I would say 99% of time it’s a miscommunication or not understanding.”	HCPs not understanding how to approach family’s lack of disease knowledge. Expecting families to make decisions without full knowledge. HCPs acknowledging that process is difficult for families is beneficial. Reminding doctors to simplify explanations.	Inadequacies of the Healthcare System
“I think some of the staff feels like well, they should be more involved in having this discussion or that discussion or involved in discharge planning, those kinds of things. I think they’re an additional support. But it’s unclear, I think for everyone what their role is, or maybe their role in that situation itself.”	Role of Indigenous support organizations is unclear/vague. Having role confusing amongst healthcare team. Blurring role lines due to high turnaround. Discussing needs of patients with team.	
“And then I think I have built some, some trusting relationships with families through the loss of their child and supporting them through that process and being there when their child is dying and then having that translate into families still reaching out after the fact. So, like being just being around it mostly involves like doing things that they need done and not, like doing what you say you will do.”	Being present to build trust. Following-up with family consistently helps with trust. Keeping consistent in interactions with families is effective for trusting relationships.	Communicating Effectively and Building Trusting Relationships
“Yeah, and just simple interactions. I think sometimes in healthcare, we overthink or we always have to go in with a mission or a reason. And we don’t, so I’m really trying to just regularly just visit with families, especially with the families that have little kids and they just need to talk to an adult. And I even try to be, try not to talk about medical stuff sometimes, I try to discuss what is, what are you doing when you’re having fun and usually that leads to something, try to relate that way.”	Trying to connect more with family. Being more present with Indigenous families. Being patient when building relationships with family.	

Colours were used to categorize the data during coding, followed by organization of the codes into broader themes.

**Table 2:** Demographic characteristics.

Healthcare Role	Number
Medical Specialist/Physician	8
Nurse/Clinical Nurse Educator	3
Social Worker	2
Pharmacist	3
Healthcare Assistant/Coordinator	1
Music Therapist	1
Registered Respiratory Therapist	1
Total	19

## Data Availability

The data are not available to the public for reasons of confidentiality.
